# Teaching Spirituality in Nursing: A Bibliometric Analysis

**DOI:** 10.1007/s10943-024-02247-6

**Published:** 2025-01-18

**Authors:** Ana Afonso, Sara Sitefane, Janaína Fabri, Isabel Rabiais, Sílvia Caldeira

**Affiliations:** 1 Faculty of Health Sciences and Nursing, Center for Interdisciplinary Research in Health, Universidade Católica Portuguesa, Palma de Cima, 1649-023 Lisbon, Portugal; 2 Faculty of Nursing, State University of Rio de Janeiro, Rio de Janeiro, Brazil; 3 Fluminense Federal University, Rio de Janeiro, Brazil; 4 Center for Interdisciplinary Research in Health, Universidade Atlântica, Higher School of Health Atlântica, 2730-036 Barcarena, Portugal

**Keywords:** Bibliometrics, Nurse, Spiritual care, Education, Teaching

## Abstract

The study of spirituality in nursing education has become an emerging academic field, making it important to understand its evolution using bibliometric indicators. To achieve this, a search was conducted on July 8, 2024, using the Web of Science and Scopus databases. Titles and abstracts were screened in Rayyan, and data analysis was performed using Bibliometrix and Biblioshiny in the R language. A total of two hundred thirty documents published between 1981 and 2024 were included. The United States contributed the most publications (*n* = 70), and Wilfred McSherry was the most prolific author, with 16 publications and the highest h-index. *Nurse Education Today* was the journal with the most publications. Transition themes identified include spiritual competence and spiritual care education.

## Introduction

Spirituality is an integral dimension of the human being. Identifying and meeting spiritual needs is an ethical imperative and central to providing holistic care. In its definition of health, relevant international organizations, such as the World Health Organization, define it not only as the absence of disease but as a dynamic state of complete physical, mental, spiritual, and social well-being (WHO, 1999). The International Council of Nurses, in its code of ethics (ICN, 2021), recognizes the importance of this human dimension.

Studies have shown the positive impact of spirituality on the healing process, the promotion of well-being, adaptation to various life situations (Ramezani et al., 2014), hope, levels of satisfaction with life, and relief from loneliness (Bulut et al., 2023). Given these benefits, nursing students and nurses must have a correct perception of spirituality and spiritual care (Wang et al., 2024) and feel competent in addressing this dimension of human care. However, a notable gap in the delivery of spiritual care is often described, mainly due to insufficient preparation and knowledge in this area (Green et al., 2020). This gap underscores the necessity of integrating spirituality more effectively into nursing curricula (Alvarenga et al., 2024).

Authors argue that the skills and attitudes to provide spiritual care can be intrinsic but also developed by teaching and learning dynamics (Ross et al., 2018). Studies show a positive association between teaching spirituality and improving knowledge, skills, and attitudes related to spiritual care (Fernández-Pascual et al., 2020), an increase in self-reported feelings of preparedness and, therefore, spiritual competence (Bush et al., 2022; Chiang et al., 2020; Green et al., 2020) as well as enhanced spiritual awareness (Chiang et al., 2020). Consequently, higher education institutions are responsible for adequately preparing nursing students and nurses to provide spiritual care.

Several pieces of evidence have been produced and written on this subject, but they continue to show this weakness in providing care. Knowing the scientific production related to the teaching of spirituality in the world will, among other things, show its evolution over time and highlight the experts in the field and the emerging themes, thus helping teachers and nursing leaders recognize the current gaps in teaching spiritual care. This is where bibliometric analysis becomes invaluable. A bibliometric review offers a systematic, transparent, reproducible approach to quantitatively analyzing scientific literature (Aria & Cuccurullo, 2017), allowing for the identification of trends, key contributors, and emerging themes within a specific field.

A preliminary search for existing bibliometric reviews on this subject has been conducted on the following databases/sources: PROSPERO and Open Science Framework. No ongoing or completed reviews specific to this care context were identified, underscoring the novelty and importance of this analysis.

## Aim of the Review

This study aims to find out about scientific production related to the teaching of spirituality in nursing by answering the following questions:

(i) How have publications on the teaching of spirituality in nursing evolved over time?
(ii) Which authors and journals have published the most on spirituality teaching in nursing?
(iii) Who are the most quoted authors on spirituality teaching in nursing?
(iv) Which countries present the highest number of publications?
(v) Which spirituality nursing teaching research topics have received the most attention in publications globally?

To this end, using bibliometrics, the following indicators will be identified in the Scopus and Web of Science databases:

- The number of publications over the years;
- The most relevant authors;
- The journals that publish the most on the subject (Bradford law);
- The authors who publish the most on the subject (Lotka law);
- The countries of publication;
- Emerging themes.

## Methods

This review was conducted according to the following inclusion criteria:

### Participants

This review considered all relevant studies considering spirituality in nursing education as objective.

### Concept

This review considered studies that address spirituality in nursing education.

### Context

This review considered all the publications on spirituality nursing education, namely all literature on undergraduate and postgraduate students.

### Type of Sources

This bibliometric review considered studies employing quantitative, qualitative, or mixed methodologies. It also considered systematic, scoping, and narrative reviews that meet the inclusion criteria.

### Search Strategy

The search strategy was developed in collaboration with UCP Libraries Reference and Research Support Service. A full search strategy was developed, including all keywords and index terms identified for each database. The search was carried out on the title, abstract and keywords using the following search equation: TITLE-ABS-KEY/TS = (Spiritual* OR “spiritual care”) AND TITLE-ABS-KEY/TS = (teach* OR educat*) AND TITLE-ABS-KEY/TS = (nurs*) AND TITLE-ABS-KEY/TS = (student* OR undergraduate OR postgraduate OR “higher education” OR baccalaureate). We used the auxiliary character asterisk to include the different word endings (Appendix).

**Fig. 1:**
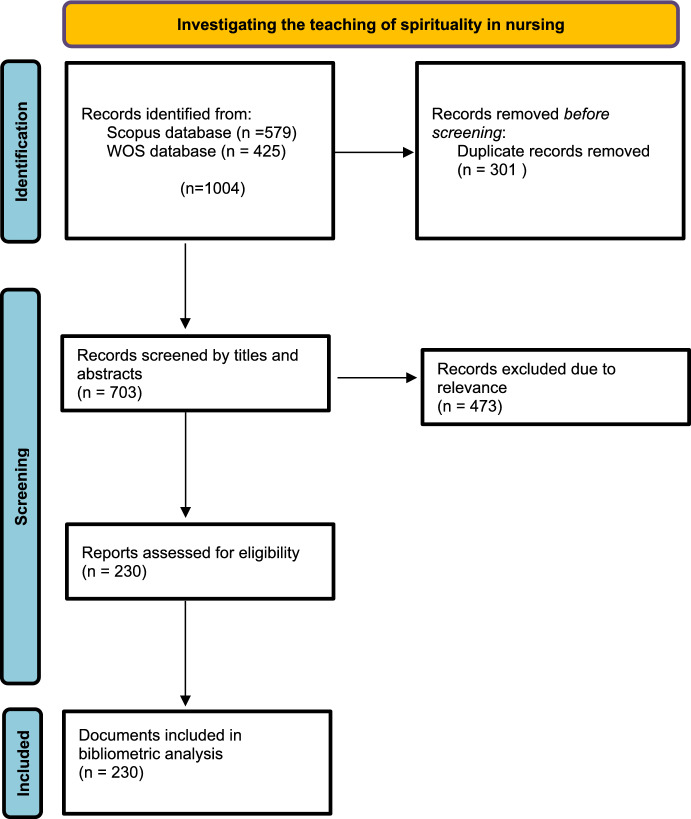
Process of selecting articles for bibliometric analysis using the PRISMA—ScR flowchart method

The search strategy was conducted on July 8, 2024, yielding a total of 1,004 results (Fig. 1).

### Study Selection

The search strategy aims to find published studies in any language. No date limit was defined for this search.

Since the databases are updated daily, the results were exported to Rayyan software on the same day. Duplicates were removed (*n* = 301), and two researchers reviewed the titles and abstracts independently. Eligible studies were then selected according to the previously mentioned criteria (*n* = 230).

### Data Extraction

The selected studies were extracted directly from the databases (Scopus and Web of Science), in the form of files compatible with the data analysis software (BibTeX—Web of Science and CSV—Scopus).

#### Data Analysis

The data were exported to the Bibliometrix software (version 4.3.0) and through RStudio (version 4.4.1). The results from the two databases were merged. This final dataset was then imported into the Biblioshiny interface, from which the various results were obtained.

## Results

Initially, 1004 documents were retrieved in the search conducted on July 8, 2024, of which 301 duplicates were excluded, resulting in 703 unique records. After a review of titles and abstracts, 230 documents were deemed eligible for the final analysis.

The first publication dates from 1981, and the annual output has been irregular over the years, as illustrated in Fig. 1. From 1999 onwards, the frequency of publications increased. In specific years, such as 2008 and 2016, we witnessed peak publication numbers, culminating in a maximum of 20 publications in 2020. Up until the current date in 2024, 11 publications have already been recorded. The data indicate an annual growth rate of 5.73%.

**Fig. 2:**
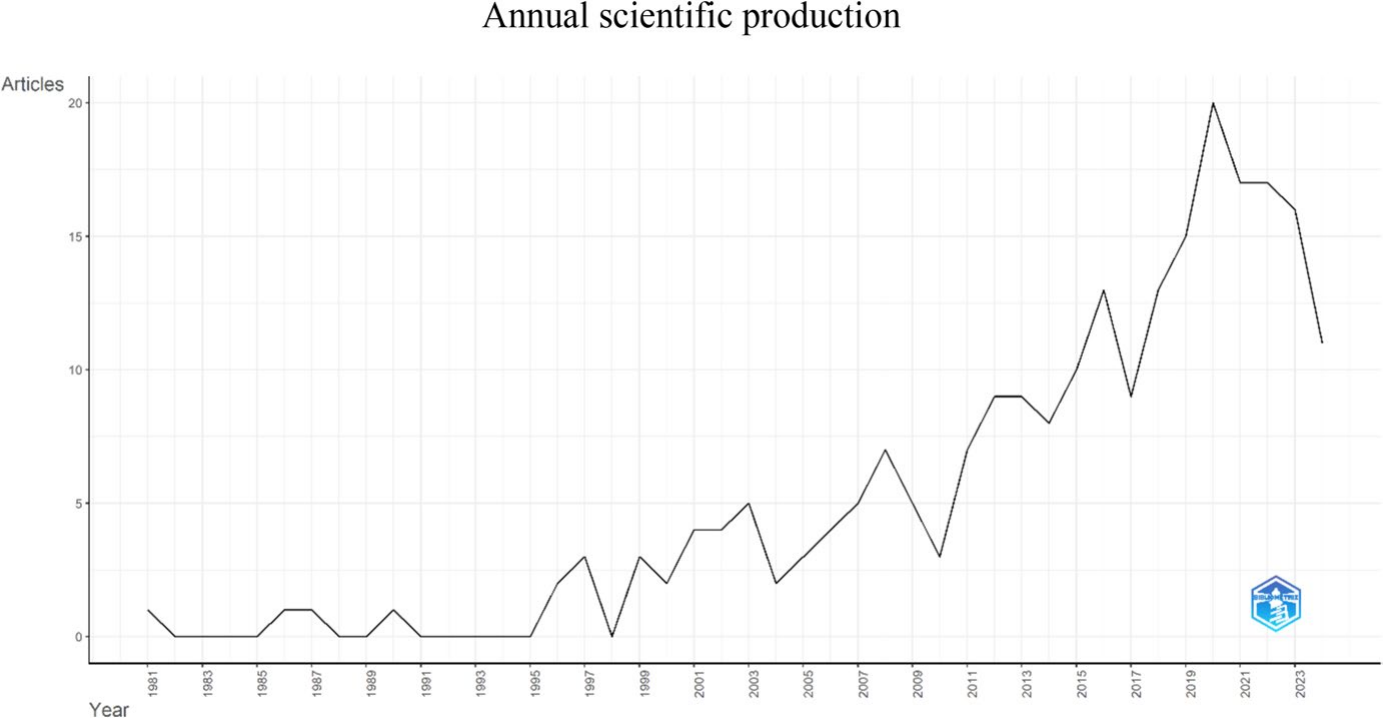
Number of publications from 1981 to 2024

Geographically, the United States leads in the number of publications, with 70 (30%), followed by the United Kingdom with 23 (10%) and Iran with 17 (7%). These three countries account for approximately 50% of the publications (Fig. 2).

**Fig. 3:**
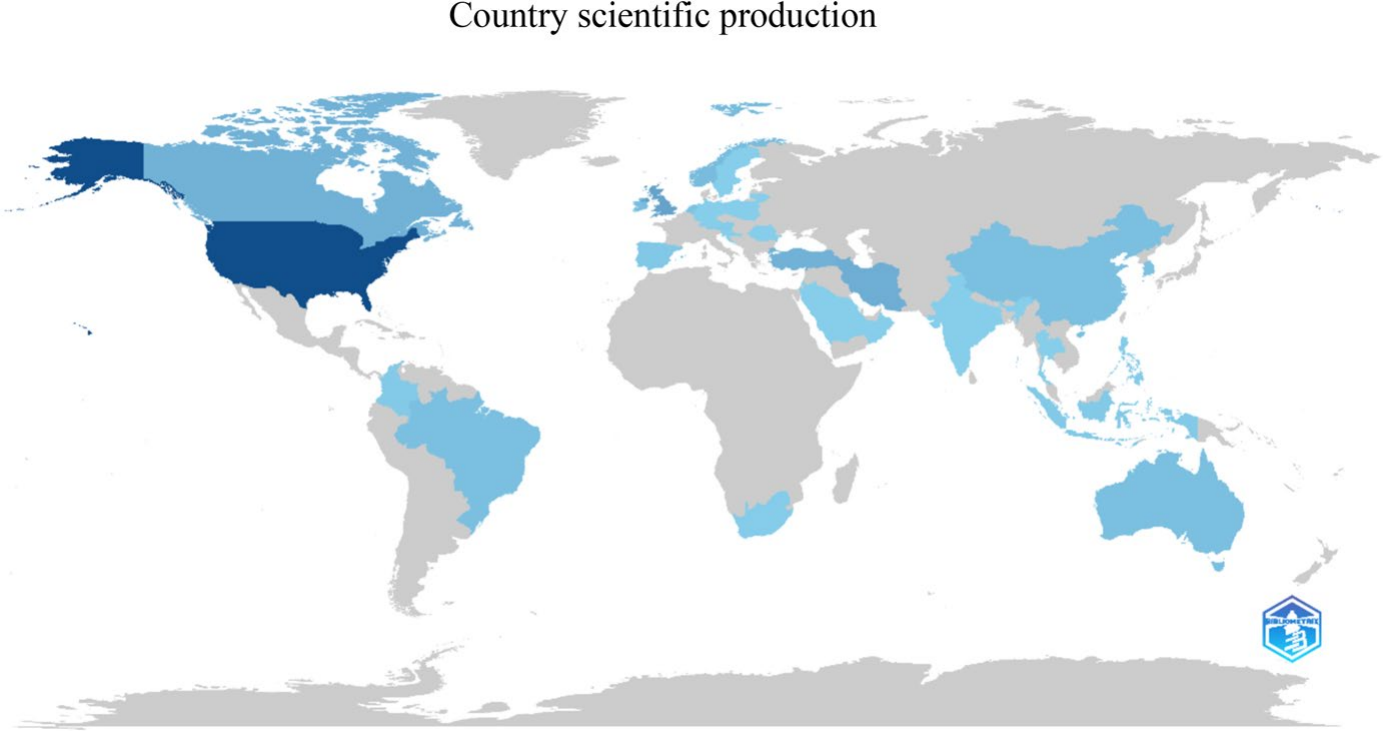
Scientific publications by country from 1981 to 2024. The countries with publications on the topic are those marked in blue on the map, the darker the blue, the higher the number of publications (Color figure online)

Among the 100 sources analyzed, the most relevant was *Nurse Education Today,* with 26 publications, followed by the *Journal of Christian Nursing: A Quarterly Publication of Nurses Christian Fellowship,* with 18 publications; *Journal of Clinical Nursing,* with 11 publications; and *Journal of Holistic Nursing* with 10 publications. In fifth place was the *Journal of Religion and Health,* with eight publications, the same as *Nurse Education in Practice* and *Nurse Educator*. Other notable sources include the *Journal of Nursing Education* and *Religions*, each with six publications, and in tenth place, *Holistic Nursing Practice,* with 5 publications, totaling 106 (46% of the total), (Fig. 3).

**Fig. 4:**
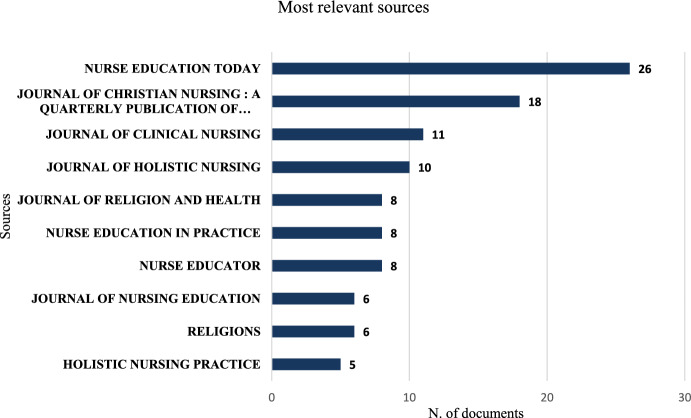
Top sources based on the number of publications

The 230 documents included contributions from 608 authors, with only 31 single-authored articles. The ten most prolific authors were Wilfred McSherry (*n* = 16), Tove Giske (*n* = 15), Linda Ross (*n* = 12), René Van Leeuwen (*n* = 11), Josephine Attard (*n* = 7), Donia Baldacchino (*n* = 7), Pamela Cone (*n* = 7), and Fiona Timmins (*n* = 7), Annemiek Schep-Akkerman (*n* = 6), and Sílvia Caldeira (*n* = 5), account for 93 publications, which is equivalent to 40% of the total (Fig. 4).

**Fig. 5:**
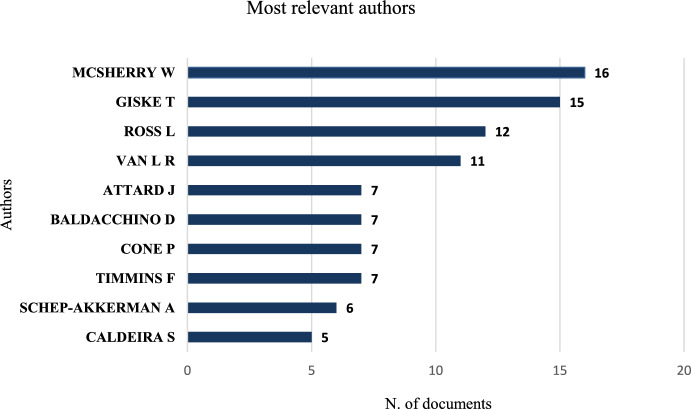
Top authors on the topic

Regarding author productivity and impact, Tove Giske and Wilfred McSherry, both with an h-index of 11, held the highest h-indexes, followed by Linda Ross with an h-index of 10, René Van Leeuwen with 9, Donia Baldacchino with 7, Josephine Attard with 6, Pamela Cone and Fiona Timmins with 5, and Adam Boughey and Aru Narayanasamy with an h-index of 4 (Fig. 5).

**Fig. 6:**
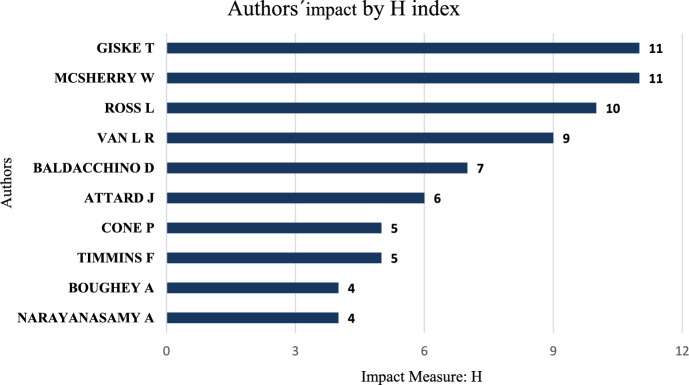
The impact of leading authors as measured by h-index

**Fig. 7:**
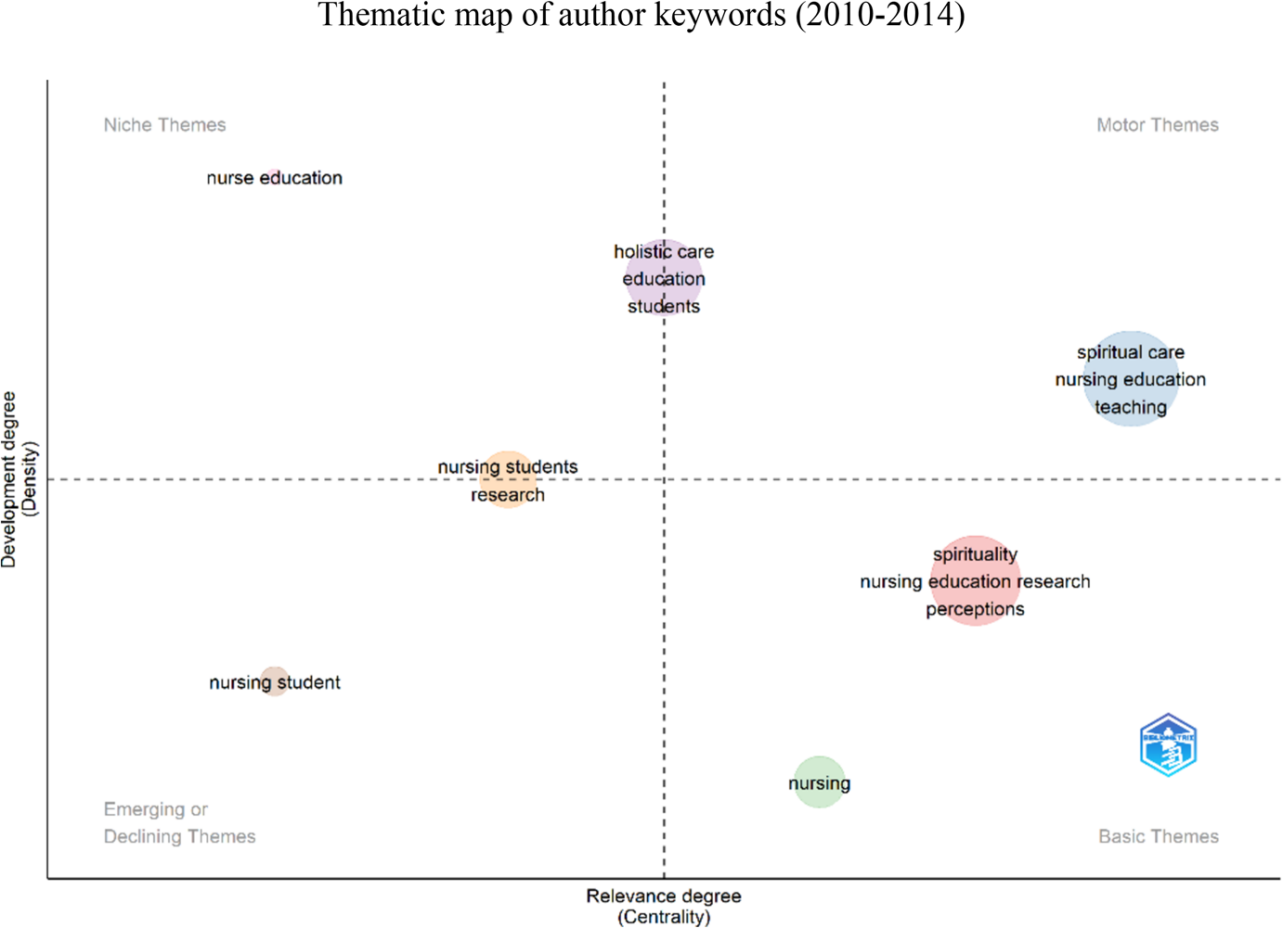
Thematic map of author keywords from 2010 to 2014

**Fig. 8:**
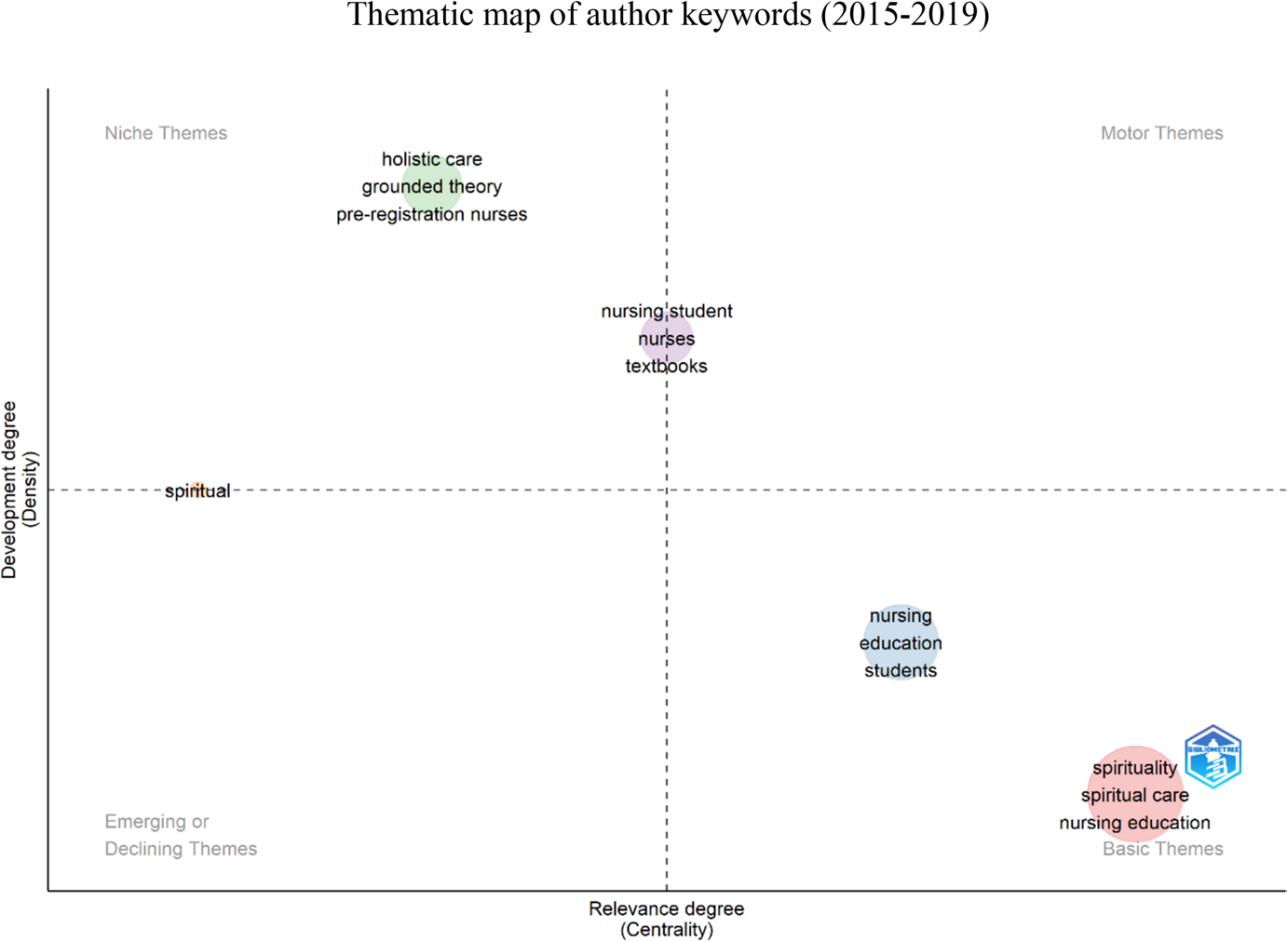
The thematic map of author keywords from 2015 to 2019

**Fig. 9:**
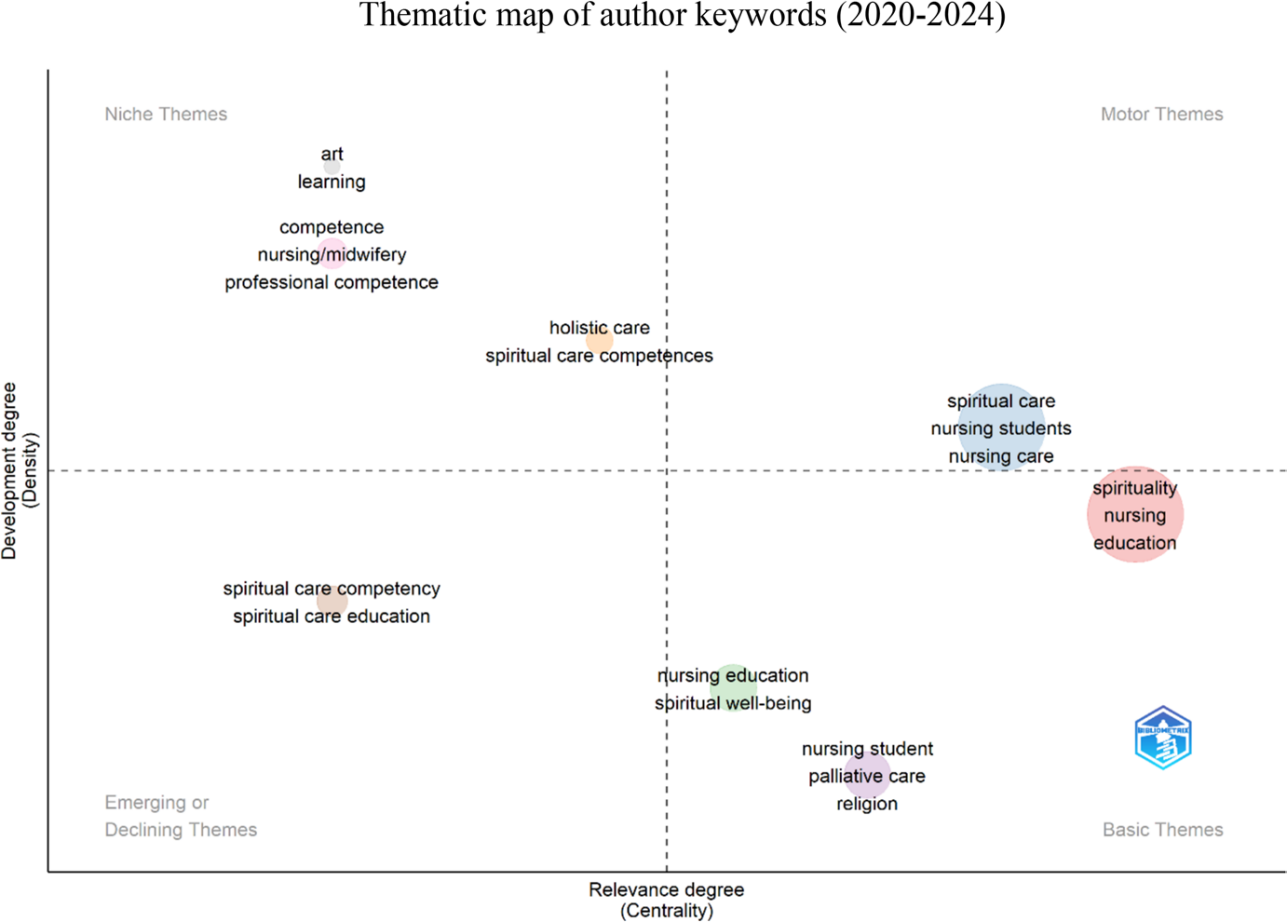
The thematic map of author keywords from 2020 to 2024

Keywords provided by the authors were utilized to create thematic maps covering three time periods: 2010–2014 (Fig. 6), 2015–2019 (Fig. 7), and 2020–2024 (Fig. 8). The aim was to explore themes' evolution and identify emerging topics. The thematic maps were represented by two main dimensions: density (internal development of the theme) and centrality (importance and influence of the theme within the research network) (Fig. 9).

During 2010–2014, high-density and centrality clusters included *spiritual care*, *nursing education*, and *teaching*. Clusters such as *spirituality*, *nursing education research*, and *perceptions* and *nursing* exhibited high centrality but low density, classified as *basic themes*. The cluster *nursing education* emerged as a *niche theme*, while the *nursing student* was identified as an *emerging or declining theme*. Other noteworthy clusters included *holistic care, education*, and *students*, positioned between *niche and motor themes, and nursing students and research positioned between niche* and *emerging or declining themes.*

In the 2015–2019 period, the clusters of *spirituality*, *spiritual care*, and *nursing education* continued to be classified as *basic themes*. Clusters such as *holistic care*, *grounded theory*, and *pre-registration nurses* were identified as *niche themes*. In contrast, *nursing students, nurses*, and *textbooks* were positioned between the quadrants of *niche themes* and *motor themes,* and between the *niche themes* and *emerging or declining themes,* there is the *spiritual* cluster.

Finally, in the 2020–2024 period, *spiritual care, nursing students*, and *nursing care* emerged as *motor themes*. The clusters of *spirituality, nursing*, and *education* were classified as *basic themes* with high proximity to *motor themes*. Other *basic themes* included *nursing students, palliative care*, and *religion*, while *nursing education* and *spiritual well-being* were close to emerging or declining themes. In the *emerging or declining themes* quadrant, clusters such as *spiritual care competence* and *spiritual care education* were identified, while *niche themes* included clusters such as *art and learning, competence, nursing/midwifery*, and *professional competence*, with *holistic care* and *spiritual care competence* positioned closer to the *motor themes* quadrant.

## Discussion

Despite not always being consistent, the development of research and publication activity in this area has shown continuous growth, with an annual growth rate of approximately 5%. This indicates a growing recognition of the importance of this topic and increased availability of updated information, which contributes to the advancement of the field.

As evidenced in Fig. 2, the first publication dates to 1981 with the article *The Spiritual Dimension of Nursing Care* [*La Dimension Spirituelle des Soins Infirmiers*]. Although this event has multiple associated factors, it can be speculated that the historical context of the spiritual dimension as an intrinsic factor in human health, documented by the WHO since 1948 and more frequently between 1978 and 1982 (Toniol, 2018), played a significant role. The inclusion of this dimension in the health concept in 1984 may also have influenced this development. Additionally, the COVID-19 pandemic, which began in 2019, may have contributed to the spike in publications in 2020 by prompting a deep reflection on human fragility and the importance of spiritual care for patients, their families, and healthcare professionals.

The United States has the highest number of publications on this topic, which may be attributed to historical, cultural, social, and political contexts. In the 2000s, there was a "spiritual awakening" in American medicine, driven by events such as the September 11, 2001, attack on the World Trade Center. This event led the country to confront identity issues and cultivate secular forms of spirituality, increasing sensitivity to beliefs and religiosity and promoting the recognition of wellness practices as an ethical principle in health (Paula, 2020). Additionally, the robustness and funding for research, as well as the recognition and advocacy for the importance of this topic by organizations like the American Nurses Association, are factors contributing to these numbers.

Funding is a crucial issue for research and publication, and countries with less financial support often face limitations in producing and disseminating knowledge. The need to pay for publishing and/or accessing updated knowledge raises an important question about each person's role in addressing this social inequity. However, Fig. 3 demonstrates publications from various countries, indicating a consensus on the importance of the topic and significant potential for international partnerships. Such partnerships can offer immense benefits by sharing updated knowledge, different countries' realities, teaching strategies, and challenges faced.

Nurse Education Today was the journal with the most publications on the topic. Founded in 1981, it is a peer-reviewed nursing journal that addresses issues in nursing, midwifery, and interprofessional health education, contributing to the advancement of nursing education. It has an impact factor of 3.6. We believe that some factors may explain this finding, namely, the journal's focus on nursing education, the fact that spirituality is an increasingly recognized dimension in holistic nursing care and that it is, therefore, appropriate for a journal dedicated to the advancement of nursing education to publish on this topic. Additionally, the journal's high impact factor enhances its appeal as a publication platform for researchers aiming to disseminate their findings.

**Fig. 10:**
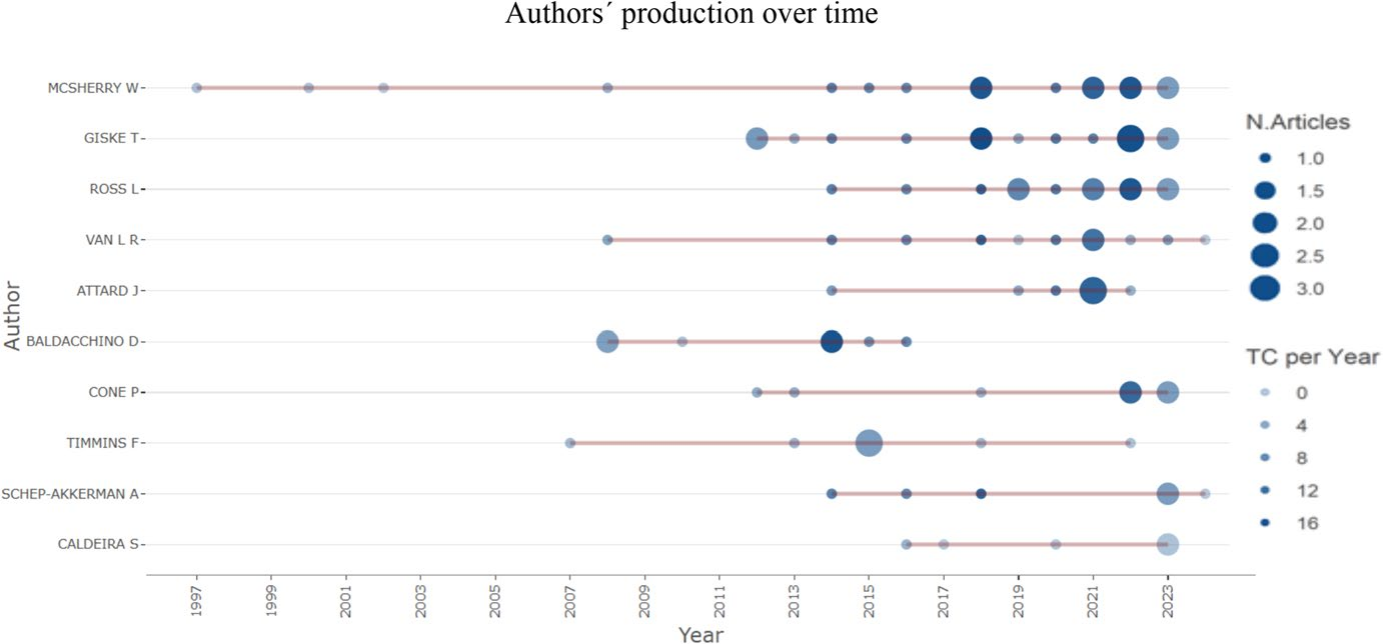
Publication of the 10 most productive authors over the years

Wilfred McSherry is identified as the most productive author. Compared to the other nine most productive authors, he began publishing on this topic in 1997 and has consistently demonstrated a steady production trajectory up to the present (Fig. 10). The bibliometric analysis revealed that although the United States and Iran lead in the number of publications on spirituality in nursing education, the most productive authors do not come from these countries. One possible explanation is that, in these countries, research may be distributed among various groups, resulting in a high volume of publications but without authors who stand out significantly in terms of productivity.

Another important point for reflection is that the most productive authors belong to the EPICC network and are concentrated in European contexts, mainly of Christian tradition. On the one hand, these findings suggest that this research group has played a fundamental role in the development of the field; on the other hand, they raise the question of whether the lack of diversity might influence the results. The homogeneity of authorship may limit the inclusion of a pluralistic view of the needs of populations from different cultural and religious contexts, affecting curriculum content and teaching practices. This could result in approaches more suitable for Christian contexts but with limitations in multicultural settings, leading to unintentional bias in students’ training and compromising their preparation to meet patients’ spiritual needs from diverse cultural backgrounds (Timmins & Neill, 2013; Wenham et al., 2021). Nevertheless, the EPICC network shows concern about this potential bias, promotes non-discriminatory education (Paal, 2024), and includes contributions from researchers from China and Kenya, who can bring different perspectives to the predominant view.

The thematic map based on author keywords is valuable because it counts keywords and groups them into clusters that reflect interrelated topics, identifying central, specialized, emerging, and marginal themes. By examining three time periods, we aimed to identify emerging topics more robustly, even if they are not the most popular. The analysis of these three periods suggests that *spiritual care*, *nursing education*, and *nursing students* remain central themes throughout, indicating their growing importance and consolidation. *Spiritual Care Competence* and *Spiritual Care Education* appear as new areas of interest in the most recent period. This may reflect a shift toward competency in handling spiritual aspects and how education can prepare nurses to address these effectively. Additionally, *art and learning* appear as a new, more specialized research trend, indicating a move toward innovative approaches in nursing education.

Furthermore, the emergence of *palliative care* and *religion* as *basic themes* in the most recent period indicates a rapid centrality without necessarily passing through the *emerging or declining* quadrant. This phenomenon could be attributed to factors such as the urgent need for responses to events like the COVID-19 pandemic or migration, emigration, and internationalization phenomena influenced by religion.

Data analysis suggests an interesting dynamic between consolidating central themes and emerging new areas of interest. The persistent focus on themes such as *Spiritual Care* and *Nursing Education* may indicate that new subfields or specializations emerge as these topics become more established. This development could signify a maturation of the discipline, where a solid foundation facilitates the exploration of novel aspects.

## Limitations

While the study provided valuable insights into thematic evolution, productivity, and the impact of authors and journals, the identified limitations suggest the need for a more holistic and diversified approach. Although we opted to use both Scopus and Web of Science for greater data robustness, differences in how these databases handle metadata—such as variations in data coverage, citation counts, and indexing practices—can introduce inconsistencies that may impact the analysis. Future proposals, such as expanding data sources, integrating qualitative analyses, and considering collaborations and regional contexts, can help provide a more comprehensive view of the research field. Exploring the most cited articles to gain deeper insights into key contributions would also be valuable.

## Conclusion

This study examined the evolution of scientific production on teaching spirituality in nursing. The key findings indicate a growing interest among researchers in this topic, driven by various historical, political, social, cultural, and health factors, as exemplified by the COVID-19 pandemic.

The study identified the most productive and influential authors in this field and the most prolific source of publications, which can serve as a valuable resource for new researchers. The thematic maps demonstrated the evolution of research topics across the analyzed periods, highlighting the transition of themes between quadrants and reflecting how research is adapting and evolving. Specialized topics are beginning to gain broader relevance.

The analysis of these maps revealed the consolidation of significant themes and the transition of new areas of interest, such as Spiritual Care Competence and Spiritual Care Education. Identifying these topics may reflect a growing awareness that, in addition to recognizing the importance of spiritual care, it is essential for professionals to be competent in this area. This trend aligns with evidence suggesting a shift in nursing *curricula* to incorporate content on spiritual care.

## Search Strategy

**Table:**

	Keywords	MeSH
Concept 1	Spirituality OR “spiritual care”	Spirituality
Concept 2	Teaching OR education	“Education, Nursing” Teaching
Concept 3	Nursing	Nursing
Concept 4	Student OR undergraduate OR postgraduate OR “higher education” OR baccalaureate	“Student, Nursing” “Education, Nurse, Graduate” “Higher education” “Education, Nursing, Baccalaureate”

## Scopus

**Table:**

	Research strategy	Results
S1	TITLE-ABS-KEY = (Spiritual* OR “spiritual care”) AND TITLE-ABS-KEY = (teach* OR educat*) AND TITLE-ABS-KEY = (nurs*) AND TITLE-ABS-KEY = (student* OR undergraduate OR postgraduate OR “higher education” OR baccalaureate)	579

## Web of Science

**Table:**

	Research strategy	Results
S1	TS = (Spiritual* OR “spiritual care”) AND TS = (teach* OR educat*) AND TS = (nurs*) AND TITLE-ABS-KEY = (student* OR undergraduate OR postgraduate OR “higher education” OR baccalaureate)	425

Research carried out on July 08th, 2024.
